# Bioinspired M-13 bacteriophage-based photonic nose for differential cell recognition†
†Electronic supplementary information (ESI) available: Instrumentation, diagrams, protein sequences and additional results. See DOI: 10.1039/c6sc02021f



**DOI:** 10.1039/c6sc02021f

**Published:** 2016-11-14

**Authors:** Jong-Sik Moon, Won-Geun Kim, Dong-Myeong Shin, So-Young Lee, Chuntae Kim, Yujin Lee, Jiye Han, Kyujung Kim, So Young Yoo, Jin-Woo Oh

**Affiliations:** a BK21 PLUS Nanoconvergence Technology Division , Pusan National University (PNU) , Busan , 46241 , Republic of Korea . Email: ojw@pusan.ac.kr; b Department of Nano Fusion Technology , Pusan National University (PNU) , Busan , 46241 , Republic of Korea; c Research Center for Energy Convergence Technology , Pusan National University (PNU) , Busan , 46241 , Republic of Korea; d Department of Cogno-Mechatronics Engineering , Pusan National University (PNU) , Busan , 46241 , Republic of Korea; e BIO-IT Foundry Technology Institute , Pusan National University (PNU) , Busan , 46241 , Republic of Korea . Email: yoosy2@gmail.com; f Research Institute for Convergence of Biomedical Science and Technology , Pusan National University (PNU) , Yangsan Hospital , Yangsan , 50612 , Republic of Korea; g Department of Nanoenergy Engineering , Pusan National University (PNU) , Busan , 46241 , Republic of Korea

## Abstract

A bioinspired M-13 bacteriophage-based photonic nose was developed for differential cell recognition. The M-13 bacteriophage-based photonic nose exhibits characteristic color patterns when phage bundle nanostructures, which were genetically modified to selectively capture vapor phase molecules, are structurally deformed. We characterized the color patterns of the phage bundle nanostructure in response to cell proliferation *via* several biomarkers differentially produced by cells, including hydrazine, *o*-xylene, ethylbenzene, ethanol and toluene. A specific color enables the successful identification of different types of molecular and cellular species. Our sensing technique utilized the versatile M-13 bacteriophage as a building block for fabricating bioinspired photonic crystals, which enables ease of fabrication and tunable selectivity through genetic engineering. Our simple and versatile bioinspired photonic nose could have possible applications in sensors for human health and national security, food discrimination, environmental monitoring, and portable and wearable sensors.

## Abstract 2

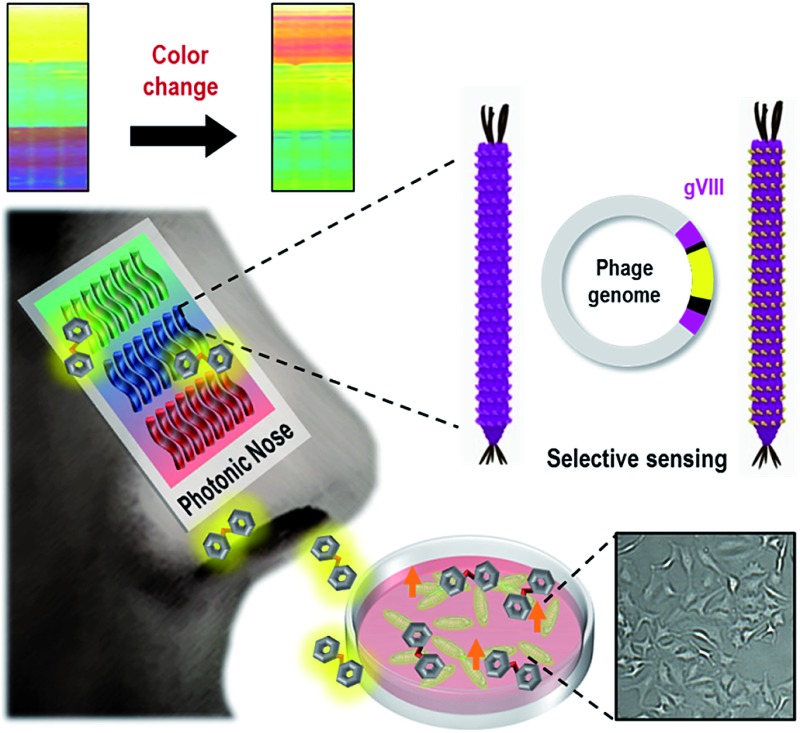
A bioinspired M-13 bacteriophage-based photonic nose was developed for differential cell recognition.

## Introduction

**Fig. 1:**
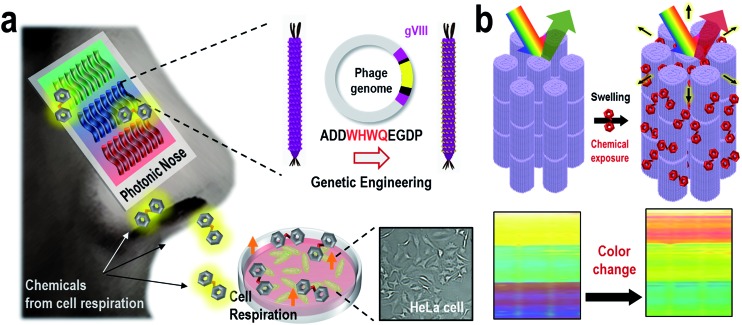
Bioinspired M-13 bacteriophage based photonic nose. (a) The cells produce specific odorants when they breathe. The M-13 bacterio-phage based photonic nose exhibits characteristic color patterns when phage bundle nanostructures, which were genetically modified to selectively capture vapor phase molecules, are structurally deformed. (b) Each phage-bundle swells or shrinks in response to vapor phase chemicals, resulting in changes in coherent scattering from the phage-bundle nanostructures.

Mimics of mammalian olfaction based on electric^1–5^ and photonic^6–10^ approaches have enabled the identification of many types of odorants in a variety of areas, including food,^11–13^ pharmaceuticals,^14,15^ security,^16,17^ space exploration^18^ and disease diagnosis.^19,20^ Artificial olfactory systems have been developed to rapidly and sensitively identify vapor phase molecules to non-invasively, inexpensively and portably monitor environmental conditions. In nature, cellular metabolism, including protein metabolism, lipid peroxidation and cholesterol biosynthesis, produces specific vapor phase compounds, some of which can be useful for both fundamental and applied research,^21^ such as metabolic pathway investigations, pathological studies and medical applications. Although odorant investigation of these metabolisms has great potential for enabling the discovery of new biomarkers and monitoring metabolic pathways, the weak and highly non-specific interactions in artificial olfactory systems have restricted the real-time monitoring of cellular metabolism. Here, we present a bioinspired M-13 bacteriophage-based photonic nose for cellular assays which monitor cellular respiration, as depicted in Fig. 1. In nature, many animals have evolved to change the colors of their skin, feathers, or exocuticle in response to external stimulation.^22–30^ For instance, turkey skin is composed of structurally organized collagen bundles, exhibiting a brilliant color due to coherent scattering from the bundle structure.^27^ Inspired by turkey skin, we fabricated an ordered M-13 bacteriophage (phage) bundle nanostructure that exhibits structural deformations on exposure to specific chemicals, leading to color changes in the resulting phage bundle. We then characterized the color patterns of the phage bundle nanostructure that occur in response to cell proliferation and several biomarkers. A specific color characteristic enables the successful identification of different types of molecular and cellular species. This approach for designing the photonic nose has great novelty in that the phage bundle nanostructure allows ease of fabrication and tunable selectivity through genetic engineering. Thus, this photonic nose based on a phage is relatively inexpensive and has lots of potential for developing a target-oriented sensor with high accuracy and high throughput.

## Results and discussion

We have employed a biomimetic, nanostructured, bacterial virus, the M-13 filamentous bacteriophage, to construct a phage-material-based photonic nose for differential cell recognition (Fig. 1a). Phages that have a well-defined nanofibrous shape and monodispersity can generate a million identical copies by infecting bacterial host cells, and each phage is composed of single-stranded DNA encapsulated by 2700 copies of the major coat protein (pVIII). The phage has previously been exploited as an advanced material for energy,^31–35^ material synthesis and assembly,^36–40^ bioengineering,^41,42^ display^43^ and sensor^44–47^ applications. The phage surface can also be easily engineered with a desired peptide for multiple functional motifs using a recombinant DNA technique, and then the engineered phage can be used in detecting target chemicals with high selectivity and sensitivity. Thus, the obvious versatility of the phage allows selective and sensitive sensing established on the biomimetic concept of using a target-specific phage bundle nanostructure rather than many cross-responsive sensor arrays.^6–9,46–54^ Peng *et al.* identified 42 volatile organic compounds from the exhaled breath of lung patients and used them as lung cancer biomarkers.^19^ In our previous study, we demonstrated WHW expressing engineered phage and used it as a color sensor for detecting TNT.^44^ WHW peptides were identified using the phage display method as specific peptides that respond to TNT vapor. Herein, we demonstrated the WHW phage bundle nanostructure and investigated its use as a color sensor for differential cell recognition by responding to differentially produced vapors from cells.

To construct the WHW–phage, we utilized a partial constrained library method to insert the desired peptides into the pVIII proteins of the M-13 phage. We attempted to express a tryptophan (W)–histidine (H)–tryptophan (W)–glutamine (Q) sequence of amino acids in the major coated protein pVIII, causing an increase in the selectivity of the aromatic hydrocarbon due to π-stacking interactions (Fig. S1†). The peptides were positioned between the first (alanine) and fifth (aspartate) amino acids of the major coat protein in a wild-type phage, replacing the EGD residues 2–4 (Ala-**Glu-Gly-Asp**-Asp to Ala-(**Desired peptides**)-Asp). For the partial library, 8 primers were designed to constrain a region of interest (*i.e.*, WHWQ) and to allow degeneracy within the flanking codons at four positions (*i.e.*, XXWHWQXX or XWHWQXXX). The favorable peptides contained both the designed motif (*i.e.*, WHWQ) as well as the unconstrained amino acid chosen by the natural selection of the *Escherichia coli* (*E. coli*) host. The relative yield and stability of the WHWQ-modified phages were verified by mass amplification, and then the sequence A*DD* **WHWQ** *EG*DP (Ala-
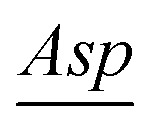
-
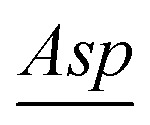
-**Trp-His-Trp-Gln**-*Glu*-
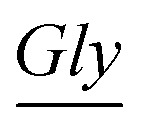
-Asp-Pro) was identified as the most stable peptide for cloning the WHWQ sequence (Fig. S2†).

**Fig. 2:**
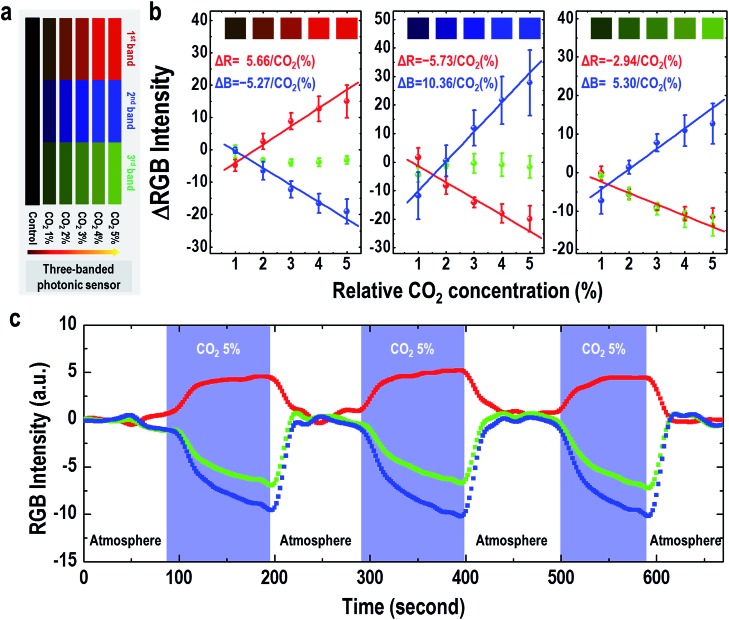
Three-banded photonic sensor for CO_2_ gas sensing. (a) Color fingerprints from the phage-based photonic nose on exposure to different carbon dioxide concentrations. (b) The RGB intensity changes in the first, second and third band of the M-13 bacteriophage-based photonic nose on exposure to different CO_2_ concentrations. (c) Reversibility of the M-13 bacteriophage-based photonic nose response to CO_2_ gas concentration.

Next, an M-13 bacteriophage-based photonic nose was fabricated using a self-templating process consisting of quasi-aligned smectic helicoidal nanofilaments with different diameters and interspacing, acting as a phage-bundle photonic crystal (Fig. S3†). The diameter and interspacing of the bundles were controlled by varying the pulling speed of the substrate. According to our previous report,^55^ the bundle diameter and interspacing decreased as the pulling speed increased. The pulling speed was varied from 20 to 40 μm min^–1^ to fabricate the red-, green- and blue-colored bands. Each phage-bundle swells or shrinks in response to external chemical gases, resulting in changes in the coherent scattering from the phage-bundles (Fig. 1b and S4†). These changes were recorded using a charge-coupled device (CCD) camera to allow precise analysis of an RGB (red, green, and blue) digital image of the phage-bundle film (Fig. S5†). We calculated the absolute RGB color level difference for each of the three-banded photonic sensors from the digital image, so that each measurement can be shown by a 3 × 3 matrix, with a total of 9 parameters. As a target chemical for monitoring cell proliferation, we have chosen carbon dioxide (CO_2_), which is one of the main components in a cell's exhalation. The virus photonic nose exhibited characteristic color changes upon exposure to different CO_2_ gas concentrations (Fig. 2a and b). The R and B values in the three-banded photonic sensor showed a linear response with increasing relative CO_2_ gas concentration (ΔR = 5.66 and ΔB = –5.27, ΔR = –5.73 and ΔB = 10.36, and ΔR = –2.94 and ΔB = 5.30 for the first, second and third band, respectively). The three-banded photonic sensor responded within 21.7 s and regained its original color within 17.9 s when we removed the stimulation, indicating that this chemical adsorption to phage-bundles is repeatable and reproducible (Fig. 2c and S6†).

**Fig. 3:**
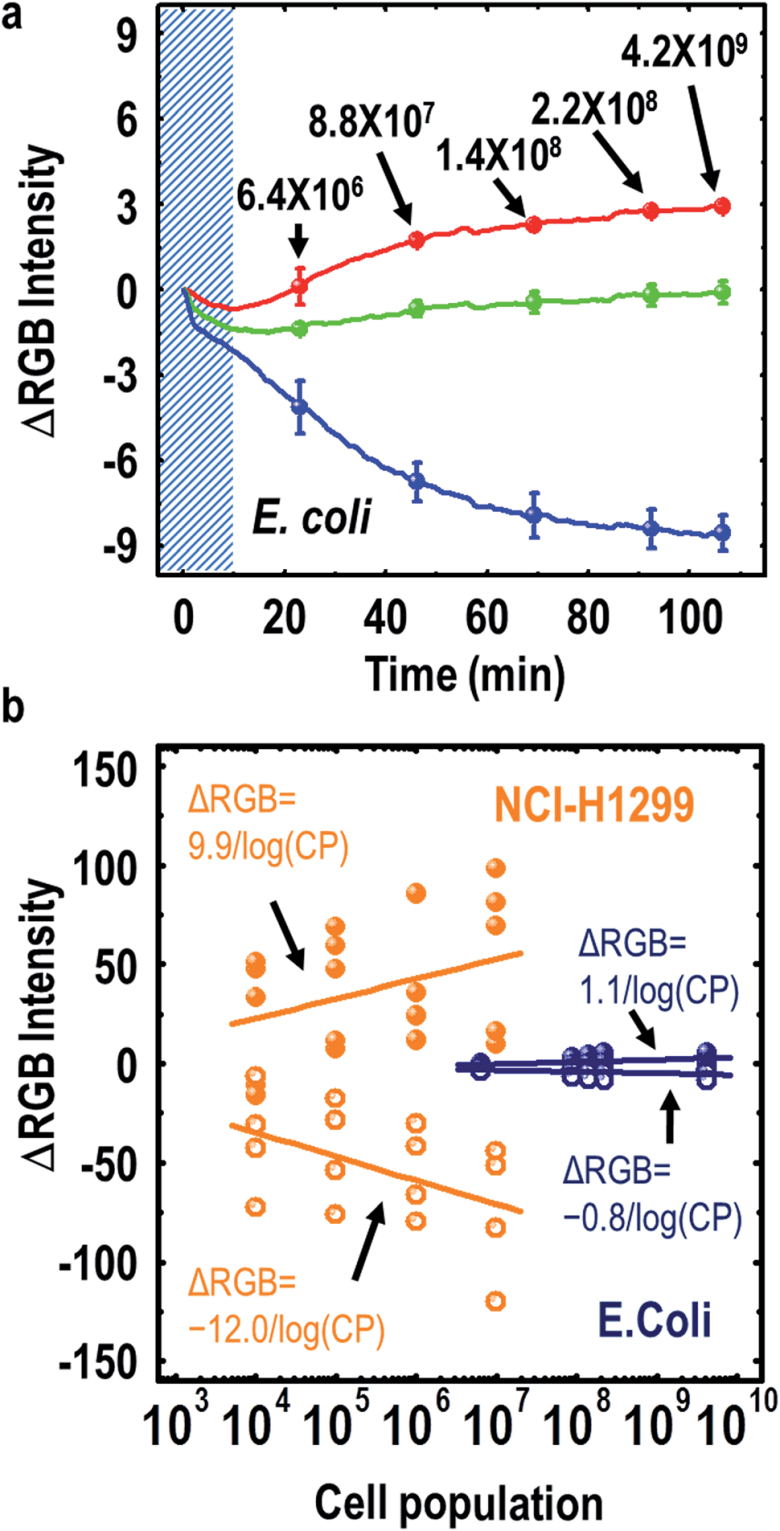
Three-banded photonic sensor for monitoring cell proliferation. (a) Real-time RGB intensity profile for *E. coli* incubated with the photonic nose. The diagonally lined area indicates the stabilization time for sensing. (b) The RGB intensity change from both *E. coli* and NCI-H1299 cells as a function of different cell populations. The R, G and B values from *E. coli* showed a linear response to cell population in a logarithmic plot (1.1/log(cell population) and –0.8/log(cell population)), and those values from NCI-H1299 cells also exhibited a linear response (9.9/log(cell population) and –12.0/log(cell population)).

First, we tested whether our M-13 bacteriophage-based photonic nose can characterize the proliferation of *E. coli* based on the response to the cell's exhalation using a three-banded photonic sensor. The B value gradually decreased with increasing cell growth time, whereas increasing the cell culture time caused the R value to increase (Fig. 3a and S7a†). We then measured the RGB values of the cell population at each time point with the optical density (*λ* = 600 nm) of the *E. coli* suspension (Fig. S7b†), and the results indicated an increased cell population with increasing cell growth time up to the highest value of 4.2 × 10^9^ cells for a culture time of 107 min. The R, G and B values (Fig. 3b) exhibited a linear response with increasing cell population in a logarithmic plot (1.1/log(cell population) and –0.8/log(cell population)). We also characterized the proliferation of NCI-H1299 lung cancer cells and obtained a linear response to the cell population (9.9/log(cell population) and –12.0/log(cell population)). The sensing capability in estimating the increase in the cell population from the color changes in the three-banded photonic sensor is, therefore, based on the amount of CO_2_ gas expelled from the cell population.

**Fig. 4:**
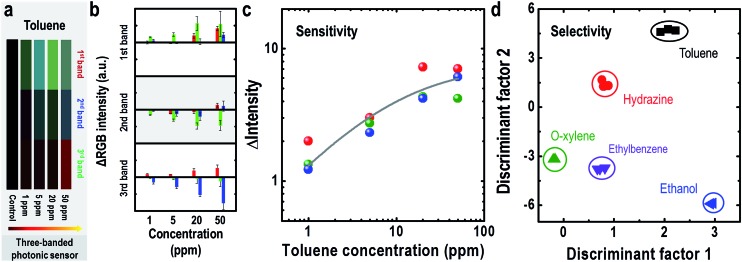
Sensitive and selective sensing using the M-13 bacteriophage based photonic sensor. Color fingerprint (a) and RGB color pattern (b) after exposure to different toluene concentrations ranging from 1 ppm to 50 ppm. (c) Sensitive sensing of the photonic nose for toluene. The data points represent each color band, and the solid line is the fitting curve from Hill's equation. The calculated dissociation constant (*K*_d_) of the complex between the phage-based photonic nose and toluene is 9.0 ppm. (d) Selective sensing of the photonic nose. Linear discriminant analysis (LDA) plot of the color changes resulting from the exposure to different vapor phase chemicals. The data in the LDA plot were taken from the color patterns of our sensor resulting from exposure to 10 ppm of toluene, hydrazine, *o*-xylene, ethanol and ethylbenzene. The vapor chemicals were applied to the sensor for 10 minutes.

Interestingly, several cells exhale characteristic aromatic chemical compounds in their respiration process, such as toluene, ethylbenzene and butylated hydroxytoluene.^19^ To demonstrate that such chemicals could be identified by the three-banded photonic sensor, our sensor was exposed to toluene, hydrazine, *o*-xylene, ethanol and ethylbenzene vapors. We used toluene as a representative chemical of cell exhalation because the WHW sequence in the major coat protein of the M-13 phage could selectively capture aromatic hydrocarbons through π–π interactions. On exposure to toluene vapor, the three-banded photonic sensor showed characteristic color changes as the toluene vapor concentration increased from 1 ppm to 50 ppm (Fig. 4a and b). The sensor responded within 67.3 s and regained its original color within 51.4 s when we removed the stimulant (Fig. S8†). From quantitative analysis using a MATLAB program for RGB color analysis, we obtained a dissociation constant (*K*_d_) of 9.0 ppm for the WHW–phage interaction with toluene (Fig. 4c). We then analyzed several chemical vapors simultaneously to identify the selectivity of our sensor (Fig S9a and b†), and presented a two-dimensional (2D) linear discriminant analysis (LDA) plot containing the results from three individual measurements (Fig. 4d). From the LDA, we obtain information about the relative importance of each discriminant factor from the variance values. The largest variance corresponds to the first discriminant factor, and the second largest one corresponds to the second discriminant factor. The value of variation in the LDA reflects the contributions of each discriminant factor to the total variance of the coordinates. We categorized the points for each of the chemical compounds, including toluene, hydrazine, *o*-xylene, ethanol and ethylbenzene, with a variance of 96%. Through such categorization of the data using LDA, we could confirm the classification capability and high reproducibility of the characteristic color change behaviors on exposure to chemical vapors.

**Fig. 5:**
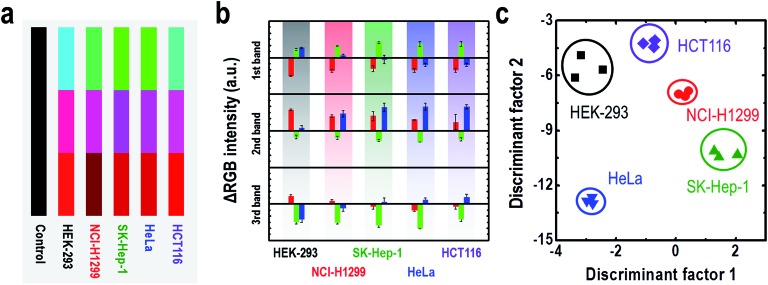
Cell type identification using the M-13 bacteriophage based photonic sensor. Color fingerprints (a) and the RGB color patterns (b) after exposure to the respiration of different cell types. (c) LDA plot of the color changes resulting from exposure to HEK-293, NCI-H1299, SKHep-1, HeLa and HCT116 cells.

To verify the feasible application of the three-banded photonic sensor, we demonstrated the potential of our platform as a cell discriminator. We selected five cell lines: human hepatic adenocarcinoma (SK-Hep-1), uterine cervical cancer (HeLa), human colon carcinoma (HCT116), human non-small lung cancer (NCI-H1299) and normal human embryonic kidney (HEK293), which were incubated in MEM containing 10% fetal bovine serum (FBS) culture medium at 37 °C in a humidified atmosphere of 5% CO_2_ in air. After growing the cells for 3 h, we mounted a new Petri dish cover with a three-banded photonic sensor attached to the inside (Fig. S4†). Because each of these cell types produces a unique composition of volatile chemical compounds, the three-banded photonic sensor develops a characteristic color, as shown in Fig. 5a (the kinetic color profiles can be seen in Fig. S10–S14†). The measurements were conducted for a set of three different well-plates of each cell, and the color patterns were identical within a tolerance of 0.03% (Fig. 5b). Fig. 5c shows the LDA plot for the cells for all measurements. This multivariate analysis method projects a 9 dimensional set of data into a 2D set of discriminant factors. The first two discriminant factors account for 99.8% of the variance of the measurements. Clustering of the data allows for all species to be reproducibly discriminated. These approaches demonstrated a rapid and effective photonic sensor for monitoring a cell’s respiration using a simple, reusable and cost-efficient platform. Additionally, this approach could possibly be further developed into a clinical alternative for cancer diagnosis and bacterial identification without the requirement of complicated and expensive methods. We believe that further development and tuning of the target-specific binding motif in phages holds promise for the development of simple and portable colorimetric sensor platforms.

## Experimental section

### Genetic modification of the M-13 phage and phage mass amplification

We genetically modified the major coat protein pVIII of the M-13 bacteriophage (New England Biolabs) using a recombinant DNA engineering technique.^41^ An inverse polymerase chain reaction (PCR) cloning method was used to replace a desired peptide sequence positioned between the first (alanine) and fifth (aspartate) amino acids of the N-terminus of the wild-type pVIII with the residues 2–4 (Ala-Glu-Gly-Asp-Asp to Ala-(Desired peptides)-Asp). We used the partial constrained library method to design a stable peptide sequence. Eight primers were prepared to constrain the region of interest (*i.e.*, WHWQ) and were unconstrained within the flanking codons at four positions (*i.e.*, XXWHWQXX or XWHWQXXX), which were chosen by the natural selection pressure of the *E. coli* host. The sequences of the products were verified *via* DNA sequencing at Cosmo Gentech (Daejeon, Republic of Korea). The genetically modified phage was amplified; the experimental details were described in our previous report.^31^

### Fabrication of the M-13 bacteriophage-based photonic nose

We fabricated the phage self-assembled color band patterns using a simple pulling method.^55^ The colors of the M-13 sensor were controlled by variation of the pulling speed between 10 and 100 μm min^–1^. To prepare the M-13 sensor, we used 6 mg mL^–1^ WHW phage suspensions in Tris-buffered saline (12.5 mM Tris and 37.5 mM NaCl, pH 7.5). Through using different pulling speeds, the M-13 sensor exhibited three perceptible color bands on gold-coated Si wafers (525 μm thickness, 100 nm of gold over a titanium adhesion layer, Platypus Co.).

### Cell monitoring

The cell suspension was placed in a culture dish (12.5 mL, SPL Co.) with MEM containing 10% FBS culture medium (Welgene Co.). In a cell culture incubator, culture dishes with cells were pre-incubated for 3 hours to obtain stable adhesion before measurement of the vapor phase chemicals. After 3 hours, the cells were washed with a PBS buffer solution and new medium was added. The CCD camera was connected with a hole on the cover of the culture dish, and data were collected every 10 seconds. A MATLAB program was run on a PC to control the camera settings.

## Conclusions

In conclusion, the bioinspired M-13 bacteriophage based photonic nose provides a straightforward, cost-efficient, sensitive and selective sensing platform for monitoring cellular metabolism. The photonic nose displays characteristic color patterns for many vapor phase molecules. A color image captured by a digital microscope allows the quantitative analysis of vapor molecules as well as the identification of different types of molecules and cellular species. Our sensing technique employed the versatile M-13 bacteriophage as a building block for fabricating biomimetic photonic crystals, enabling ease of fabrication and tunable selectivity through genetic engineering. We believe that our simple and versatile M-13 bacteriophage-based photonic nose could have feasible applications in food discrimination, environmental monitoring and portable and wearable sensors. Also, the photonic nose can be usefully applied to the design of sensors for human health and national security.

## References

[cit1] Persaud K., Dodd G. (1982). Nature.

[cit2] Lundstrom I. (2000). Nature.

[cit3] Baik J. M., Zielke M., Kim M. H., Turner K. L., Wodtke A. M., Moskovits M. (2010). ACS Nano.

[cit4] Ko W. R., Jung N. C., Lee M. C., Yun M. H., Jeon S. M. (2013). ACS Nano.

[cit5] Kwon O. S., Song H. S., Park E. J., Lee S. H., An J. H., Park J. W., Ynag H., Yoon H., Bae J., Park T. H., Jang J. (2015). Nano Lett..

[cit6] Bonifacio L. D., Puzzo D. P., Breslav S., Willey B. M., McGeer A., Ozin G. A. (2010). Adv. Mater..

[cit7] Lotsch B. V., Ozin C. A. (2008). Adv. Mater..

[cit8] Redel E., Mirtchev P., Huai C., Petrov S., Ozin G. A. (2011). ACS Nano.

[cit9] Lim S. H., Feng L., Kemling J. W., Musto C. J., Suslick K. S. (2009). Nat. Chem..

[cit10] Xie Z., Cao K., Zhao Y., Bai L., Gu H., Xu H., Gu Z. Z. (2014). Adv. Mater..

[cit11] Tao H., Brenckle M. A., Yang M., Jhang J., Liu M., Siebert S. M., Averitt R. D., Mannoor M. S., McAlpine M. C., Rogers J. A., Kaplan D. L., Omenetto F. G. (2012). Adv. Mater..

[cit12] Zhang C., Suslick K. S. (2005). J. Am. Chem. Soc..

[cit13] Lim S. H., Musto C. J., Park E., Zhong W., Suslick K. S. (2008). Org. Lett..

[cit14] Zhu L., Seburg R. A., Tsai E., Puech S., Mifsud J. C. (2004). J. Pharm. Biomed. Anal..

[cit15] Baldwin E. A., Bai J., Plotto A., Dea S. (2011). Sensors.

[cit16] Feng L., Musto C. J., Kemling J. W., Lim S. H., Suslick K. S. (2010). Chem. Commun..

[cit17] Feng L., Musto C. J., Kemling J. W., Lim S. H., Zhong W., Suslick K. S. (2010). Anal. Chem..

[cit18] Krabach T., Aerosp. Conf. Prof., 2000, vol. 6, p. 565.

[cit19] Peng G., Tisch U., Adams O., Hakim M., Shehada N., Broza Y. Y., Billan S., Bortnyak R. A., Kuten A., Haick H. (2009). Nat. Nanotechnol..

[cit20] Bardhan N. M., Ghosh D., Belcher A. M. (2014). Nat. Commun..

[cit21] Kalluri U., Naiker M., Myers M. A. (2014). J. Breath Res..

[cit22] Kolle M., Salgard-Cunha P.
M., Scherer M. R. J., Huang F., Vukusic P., Mahajan S., Baumberg J. J., Steiner U. (2010). Nat. Nanotechnol..

[cit23] Vukusic P., Sambles J. R. (2003). Nature.

[cit24] Sharma V., Crne M., Park J. O., Srinivasarao M. (2009). Science.

[cit25] Kramer R. M., Crookes-Goodson W. J., Naik R. R. (2007). Nat. Mater..

[cit26] Prum R. O., Torres R. H. (2004). J. Exp. Biol..

[cit27] Prum R. O., Torres R. H. (2003). J. Exp. Biol..

[cit28] Kinoshita S., Yoshioka S. (2005). ChemPhysChem.

[cit29] Noh H., Liew S. F., Saranathan V., Mochrie S. G. J., Prum R. O., Dufresne E. R., Cao H. (2010). Adv. Mater..

[cit30] Forster J. D., Noh H., Liew S. F., Saranathan V., Schreck C. F., Yang L., Park J.-G., Prum R. O., Mochrie S. G. J., O'Hern C. S., Cao H., Dufresne E. R. (2010). Adv. Mater..

[cit31] Shin D. M., Han H. J., Kim W. G., Kim E., Kim C., Hong S. W., Kim H. K., Oh J. W., Hwang Y. H. (2015). Energy Environ. Sci..

[cit32] Lee B. Y., Zhang J., Zueger C., Chung W. J., Yoo S. Y., Wang E., Meyer J., Ramesh R., Lee S. W. (2012). Nat. Nanotechnol..

[cit33] Oh D. H., Qi J., Han B. H., Zhang G., Carney T. J., Ohmura J., Zhang Y., Shao-Horn Y., Belcher A. M. (2015). Nano Lett..

[cit34] Lee Y. J., Yi H., Kim W. J., Kang K., Yun D. S., Strano M. S., Ceder G., Belcher A. M. (2009). Science.

[cit35] Mao C., Solis D. J., Reiss B. D., Kottmann S. T., Sweeney R. Y., Hayhurst A., Georgiou G., Iverson B., Belcher A. M. (2004). Science.

[cit36] Nam K. T., Kim D. W., Yoo P. J., Chiang C. Y., Meethong N., Hammond P. T., Chiang Y. M., Belcher A. M. (2006). Science.

[cit37] Chen P., Hyder M. N., Mackanic D., Courchesne N. D., Qi J., Klug M. T., Belcher A. M., Hammond P. T. (2014). Adv. Mater..

[cit38] Courchesne N. D., Klung M. T., Chen P., Kooi S. E., Yun D. S., Hong N., Fang N. X., Belcher A. M., Hammond P. T. (2014). Adv. Mater..

[cit39] Lee S. W., Mao C., Flynn C. E., Belcher A. M. (2002). Science.

[cit40] Wang J., Yang M., Zhu Y., Wang L., Tomsia A. P., Mao C. (2014). Adv. Mater..

[cit41] Merzlyak A., Lee S.-W. (2009). Bioconjugate Chem..

[cit42] Bhattarai S. R., Yoo S. Y., Lee S. W., Dean D. (2012). Biomaterials.

[cit43] Nam K. T., Peelle B. R., Lee S. W., Belcher A. M. (2004). Nano Lett..

[cit44] Oh J. W., Chung W. J., Heo K., Jin H. E., Lee B. Y., Wang E., Zueger C., Wong W., Meyer J., Kim C. T., Lee S. Y., Kim W. G., Zemla M., Auer M., Hexemer A., Lee S. Y. (2014). Nat. Commun..

[cit45] Yoo S. Y., Oh J. W., Lee S. W. (2012). Langmuir.

[cit46] Zhou X., Cao P., Zhu Y., Lu W., Gu N., Mao C. (2015). Nat. Mater..

[cit47] Wang Y., Ju Z., Cao B., Gao X., Zhu Y., Qiu P., Xu H., Pan P., Bao H., Wang L., Mao C. (2015). ACS Nano.

[cit48] Rakow N. A., Suslick K. S. (2000). Nature.

[cit49] Janzen M. C., Ponder J. B., Bailey D. P., Ingison C. K., Suslick K. S. (2006). Anal. Chem..

[cit50] Lin H., Jang M., Suslick K. S. (2011). J. Am. Chem. Soc..

[cit51] Feng L., Musto C. J., Suslick K. S. (2010). J. Am. Chem. Soc..

[cit52] Carey J. R., Suslick K. S., Hulkower K. I., Imlay J. A., Imlay K. R. C., Ingison C. K., Ponder J. B., Sen A., Wittrig A. E. (2011). J. Am. Chem. Soc..

[cit53] Moon J. S., Park M. J., Kim W. G., Kim C. T., Hwang J. Y., Seol D., Kim C. S., Sohn J. R., Chung H., Oh J. W. (2017). Sens. Actuat. B.

[cit54] Moon J. S., Lee Y. J., Shin D. M., Kim C. T., Kim W. G., Park M. J., Han J. Y., Song H. R., Kim K. J., Oh J. W. (2016). Chem. Asian J..

[cit55] Chung W. J., Oh J. W., Kwak K., Lee B. Y., Meyer J., Wang E., Hexemer A., Lee S. W. (2011). Nature.

