# Correction: Bioinspired M-13 bacteriophage-based photonic nose for differential cell recognition

**DOI:** 10.1039/c6sc90081j

**Published:** 2016-12-13

**Authors:** Jong-Sik Moon, Won-Geun Kim, Dong-Myeong Shin, So-Young Lee, Chuntae Kim, Yujin Lee, Jiye Han, Kyujung Kim, So Young Yoo, Jin-Woo Oh

**Affiliations:** a BK21 PLUS Nanoconvergence Technology Division , Pusan National University (PNU) , Busan , 46241 , Republic of Korea . Email: ojw@pusan.ac.kr; b Department of Nano Fusion Technology , Pusan National University (PNU) , Busan , 46241 , Republic of Korea; c Research Center for Energy Convergence Technology , Pusan National University (PNU) , Busan , 46241 , Republic of Korea; d Department of Cogno-Mechatronics Engineering , Pusan National University (PNU) , Busan , 46241 , Republic of Korea; e BIO-IT Foundry Technology Institute , Pusan National University (PNU) , Busan , 46241 , Republic of Korea . Email: yoosy2@gmail.com; f Research Institute for Convergence of Biomedical Science and Technology , Pusan National University (PNU) , Yangsan Hospital , Yangsan , 50612 , Republic of Korea; g Department of Nanoenergy Engineering , Pusan National University (PNU) , Busan , 46241 , Republic of Korea

## Abstract

Correction for ‘Bioinspired M-13 bacteriophage-based photonic nose for differential cell recognition’ by Jong-Sik Moon *et al.*, *Chem. Sci.*, 2017, DOI: 10.1039/c6sc02021f.



## 

The authors regret that a funding acknowledgement was omitted from the original article. A revised version of the Acknowledgements section, in which funding from the Ministry of Health & Welfare, Republic of Korea, is now acknowledged, is included herein.

The Royal Society of Chemistry apologises for these errors and any consequent inconvenience to authors and readers.

