# Supplementary material for: Bioinspired M-13 bacteriophage-based photonic nose for differential cell recognition
Source: Chem Sci. 2016 Nov 14;8(2):921–7. doi: 10.1039/c6sc02021f (PMC5452260; doi:10.1039/c6sc02021f)
Supplement: Supplementary file 1 [file SC-008-C6SC02021F-s001.pdf]

Electronic Supplementary Material (ESI) for Chemical Science.
This journal is © The Royal Society of Chemistry 2016

Supporting Information for

Bioinspired M-13 bacteriophage-based Photonic Nose for Differential Cell Recognition

Jong-Sik Moon[a,†], Won-Geun Kim[a,b,†], Dong-Myeong Shin[c,†], So-Young Lee[a,b], Chuntae Kim[a,b], Yujin Lee[a,b], Jiye Han[a,b], Kyujung Kim[d], So Young Yoo[e,f,] and Jin-Woo Oh[a,b,g,]**

[a] BK21 PLUS Nanoconvergence Technology Division, Pusan National University (PNU), Busan, 46241, Republic of Korea

[b] Department of Nano Fusion Technology, Pusan National University (PNU), Busan, 46241, Republic of Korea

[c] Research Center for Energy Convergence Technology, Pusan National University (PNU), Busan, 46241, Republic of Korea

[d] Department of Cogno-Mechatronics Engineering, Pusan National University (PNU), Busan, 46241, Republic of Korea

[e] BIO-IT Foundry Technology Institute, Pusan National University (PNU), Busan, 46241, Republic of Korea

[f] Research Institute for Convergence of Biomedical Science and Technology, Pusan National University (PNU) Yangsan Hospital, Yangsan, 50612, Republic of Korea

[g] Department of Nanoenergy Engineering, Pusan National University (PNU), Busan, 46241, Republic of Korea

KEYWORDS Photonic nose, Biomimetic photonic crystal, Target-specific sensing, M-13 bacteriophage, Cellular respiration

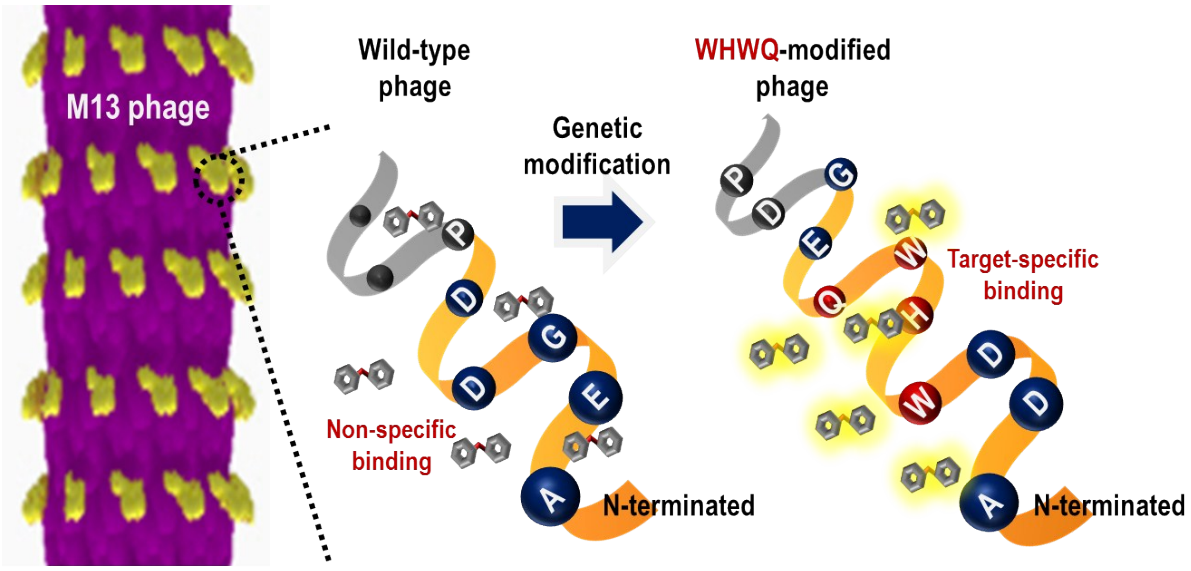

Figure S1. Schematic representation of a single pVIII major coated protein that is genetically modified to selectively detect aromatic hydrocarbon molecules for target-specific sensing.

a

Sequence#	pVIII WHWQ sequences										
1	A	D	D	W	H	W	Q	E	G	D	P
2	A	D	D	W	H	W	Q	D	G	D	P
3	A	E	D	W	H	W	Q	D	G	D	P
4	A	E	E	W	H	W	Q	E	G	D	P
5	A	E	E	W	H	W	Q	E	S	D	P
6	A	D	T	W	H	W	Q	E	E	D	P
7	A	D	W	H	W	Q	E	S	E	D	P

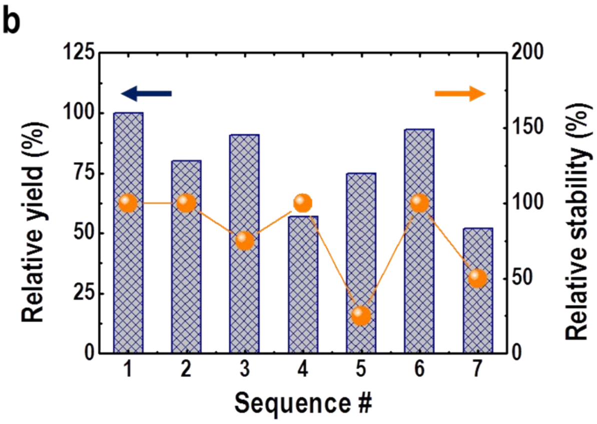

Figure S2. (a) WHWQ constrained library sequences. (b) Relative yield and stability of the engineered peptide sequences.

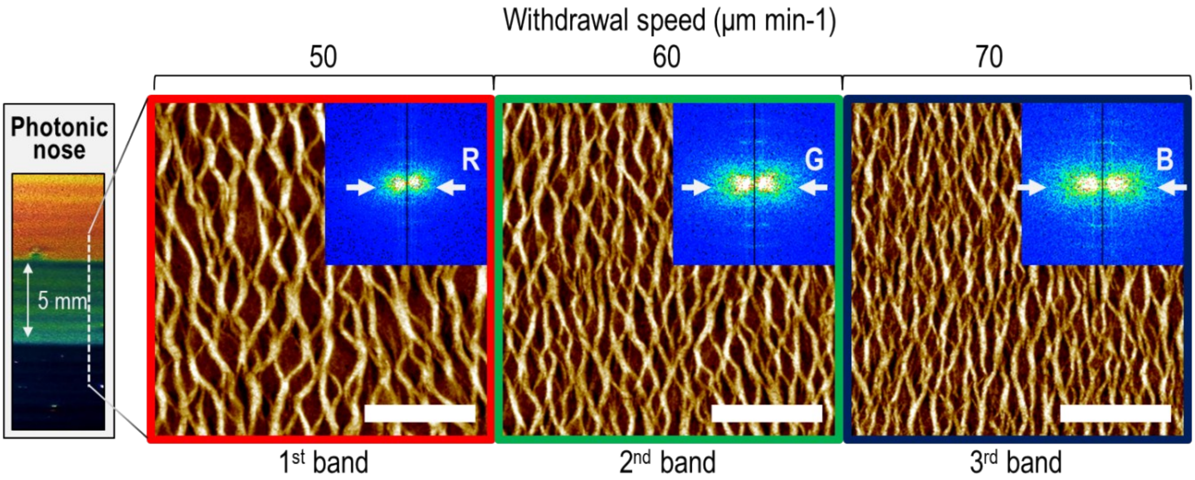

Figure S3. Photograph and atomic force microscope (AFM) images of the photonic nose. Each matrix is composed of an ordered phage bundle nanostructure with different diameters and interspacing. Insets indicate the two-dimensional fast Fourier transform (FFT) images of the 1st band (red), 2nd band (green) and 3rd band (blue). Scale bar=10 μm.

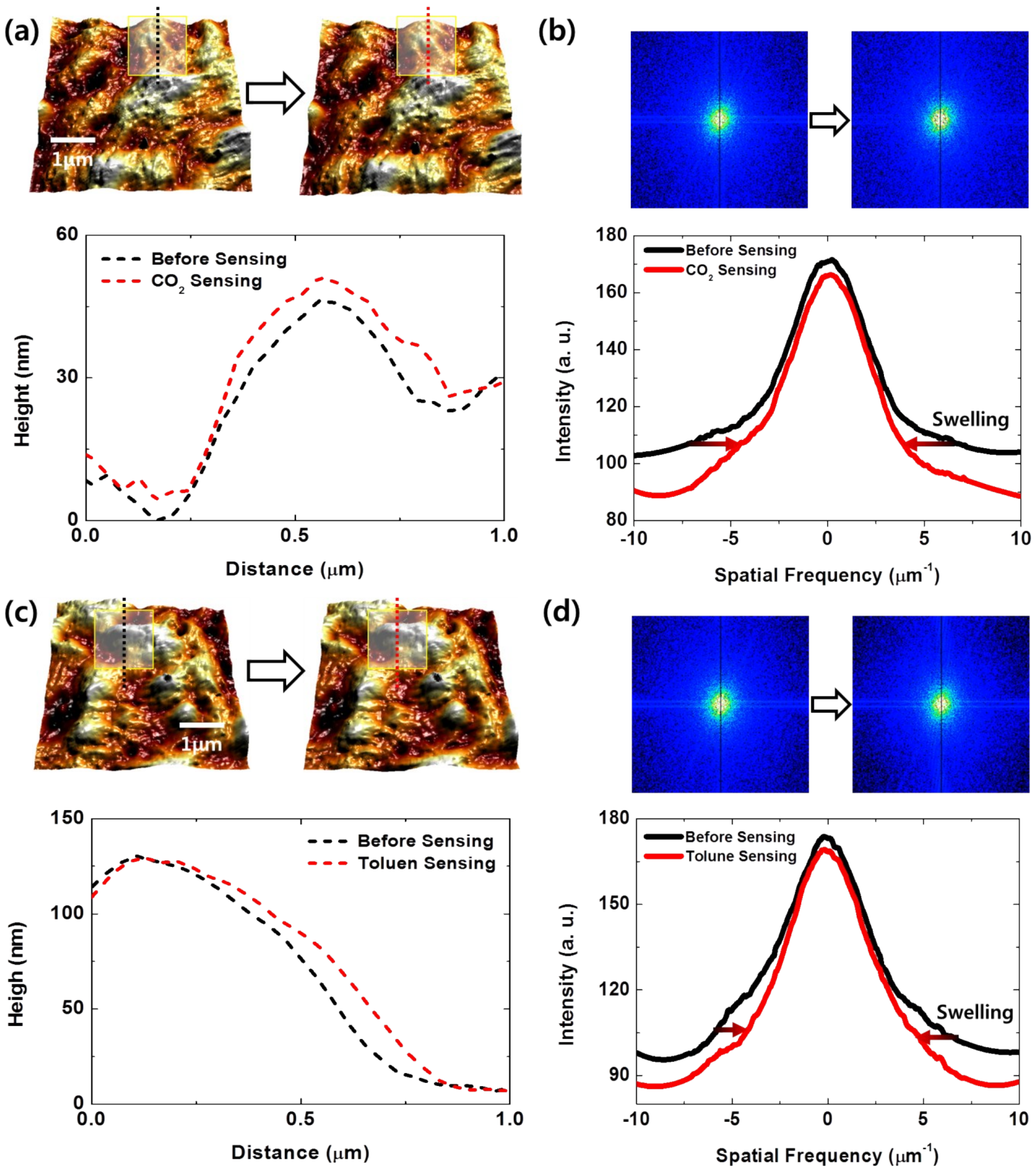

Figure S4. (a) AFM image set of a self-assembled M13 phage nanostructure before and after CO_2 exposure (b) Fast Fourier transform (FFT) analysis of the AFM image before and after CO_2 exposure (c) AFM image set of a self-assembled M13 phage nanostructure before and after toluene exposure (d) FFT analysis of the AFM image before and after toluene exposure.

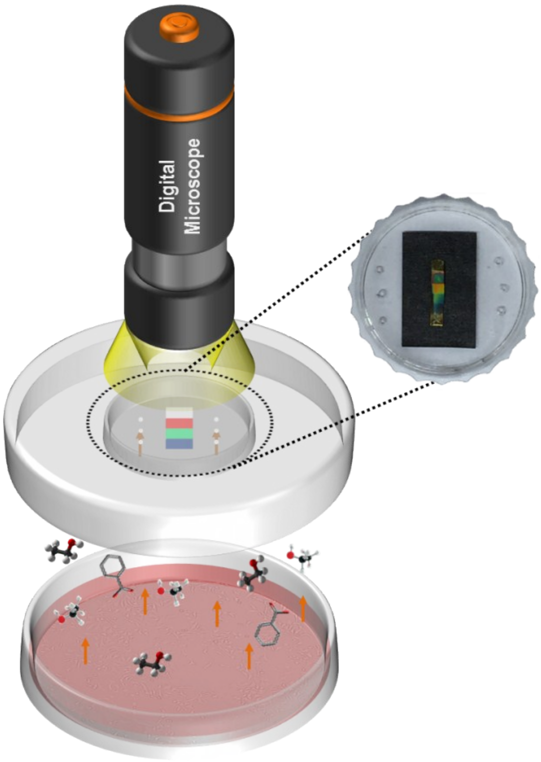

Figure S5. Schematic illustration of the experimental setup for monitoring cell respiration. The photonic nose was placed in the petri dish where cells were incubated, and then the digital color image was captured by microscope.

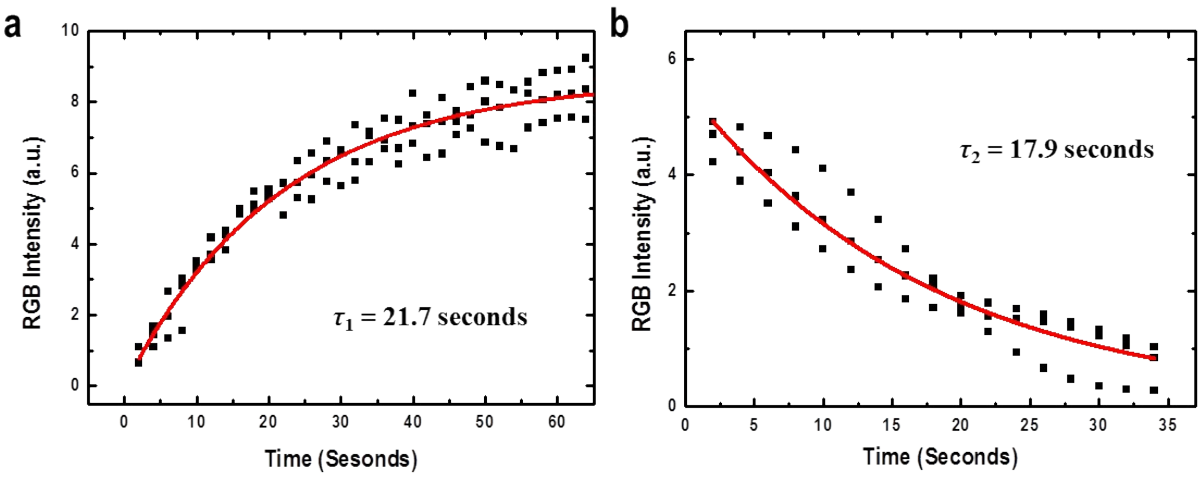

Figure S6. The response time of three-banded photonic sensor on exposure to 5% of CO_2 gas. The time constants, τ_1 *and* τ_2 were calculated by fitting the evolution of the growth and decrease of the color intensity, *i(t)*, with the following exponential function. $i(t) = a\{1 - exp(-t/\tau_1)\}$ and $i(t) = a\{exp(-t/\tau_2)\}$.

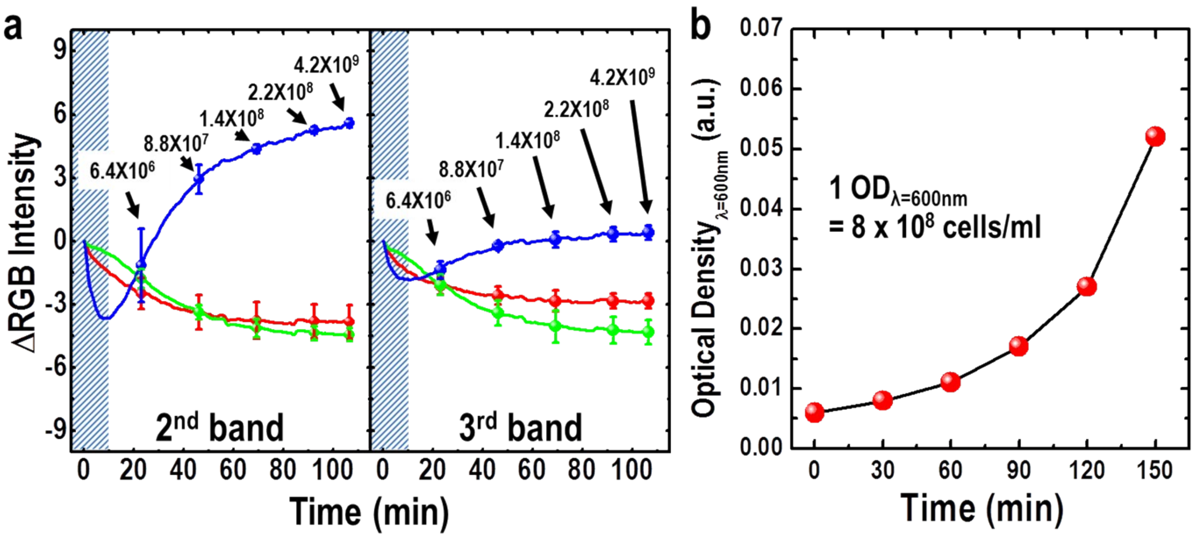

Figure S7. (a) Real-time RGB intensity profiles in the second and third band of the M-13 bacteriophage-based photonic nose while *E. coli* was incubated. The diagonally-across-marked area indicates the stabilization time for sensing. (b) The optical density (OD) profile for calculating the *E. coli* population where 1 $OD_{\lambda=600nm}$=8 X 10^8 cells/ml.

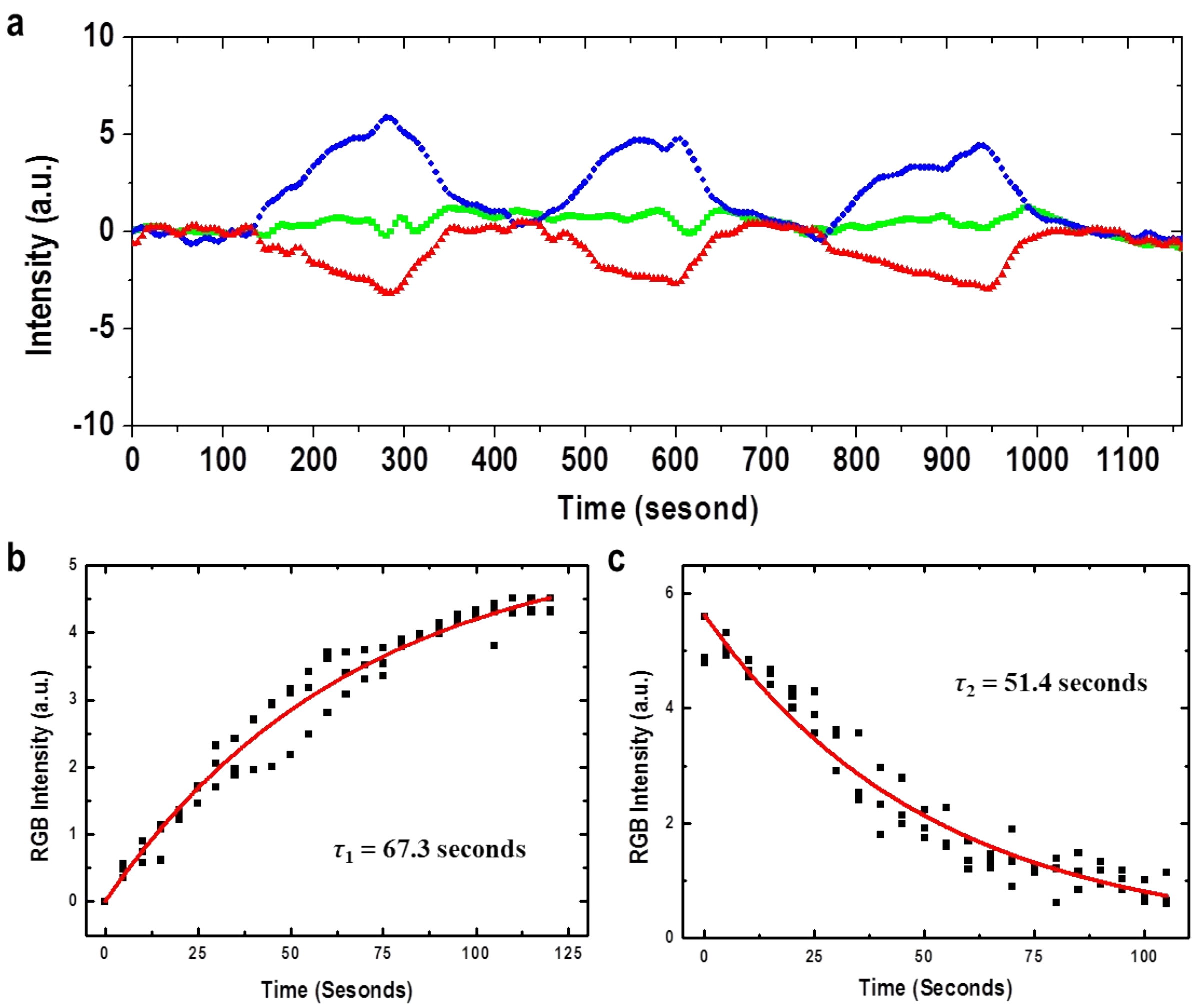

Figure S8. Reversibility of M-13 bacteriophage color sensor upon exposure to toluene.

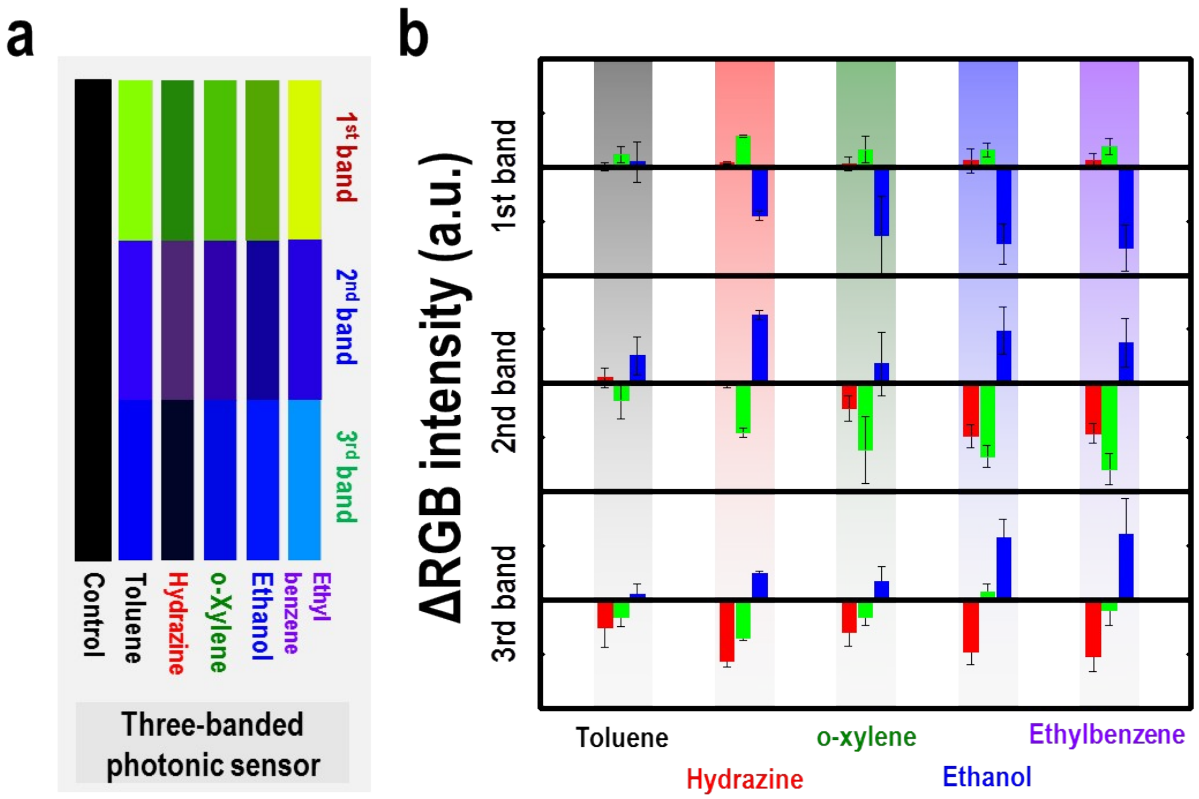

Figure S9. Color fingerprint (a) and RGB color pattern (b) used to selectively distinguish various chemicals.

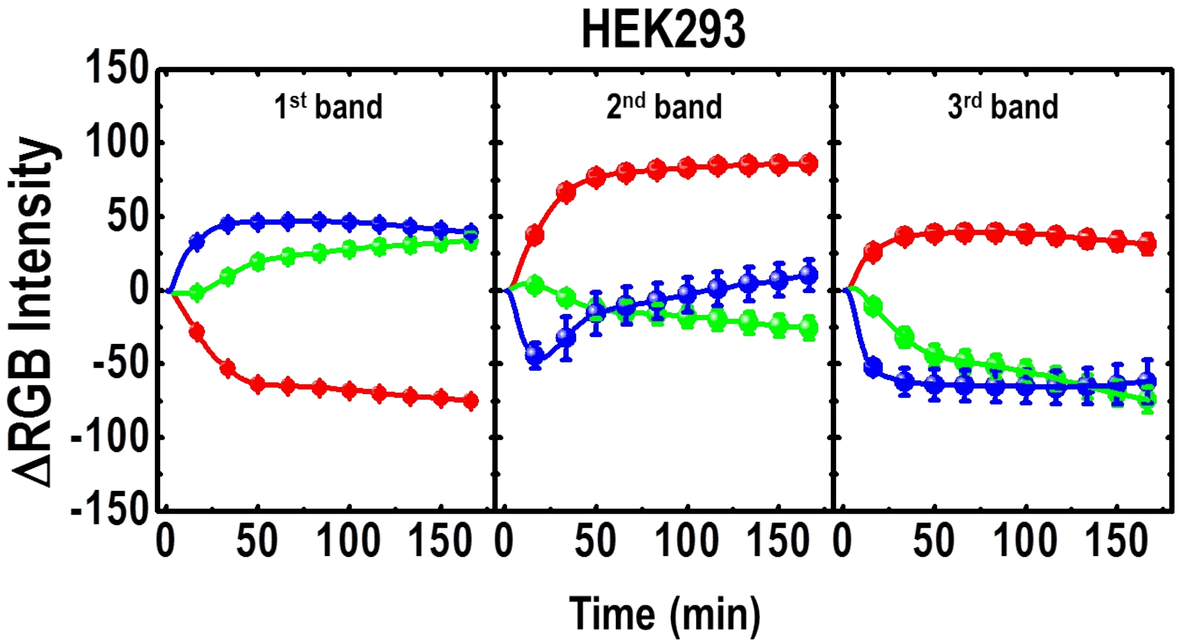

Figure S10. Real-time RGB color profiles of the M-13 bacteriophage-based photonic nose after exposure to HEK293 cell proliferation.

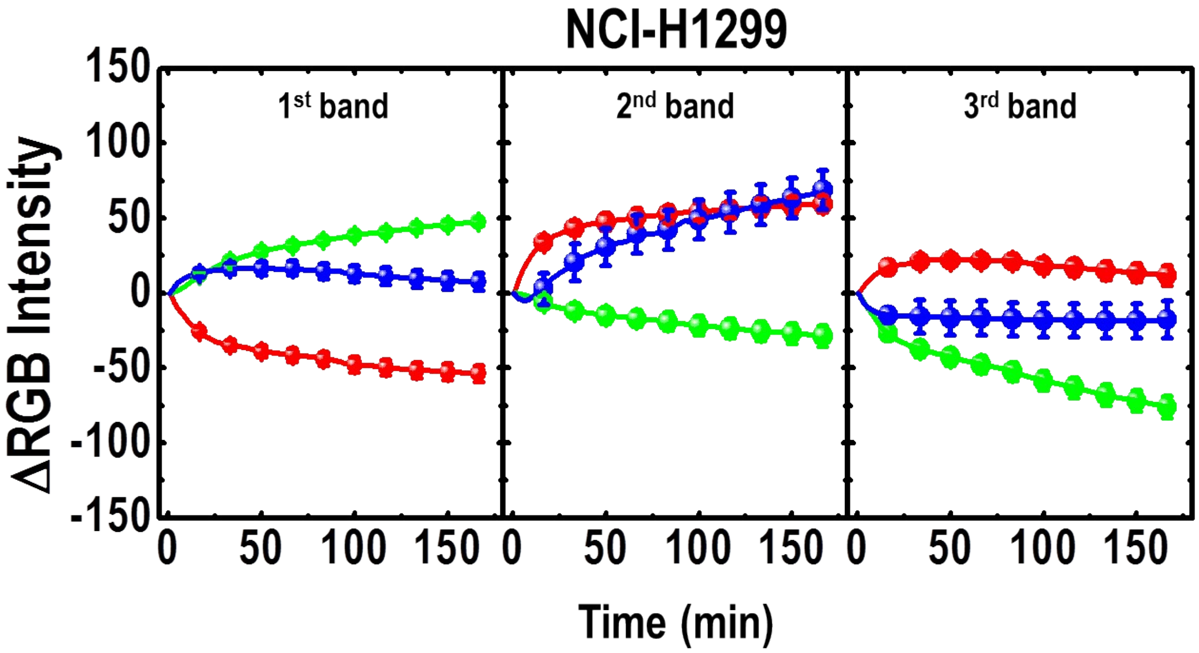

Figure S11. Real-time RGB color profiles of the M-13 bacteriophage-based photonic nose after exposure to NCI-H1299 cell proliferation.

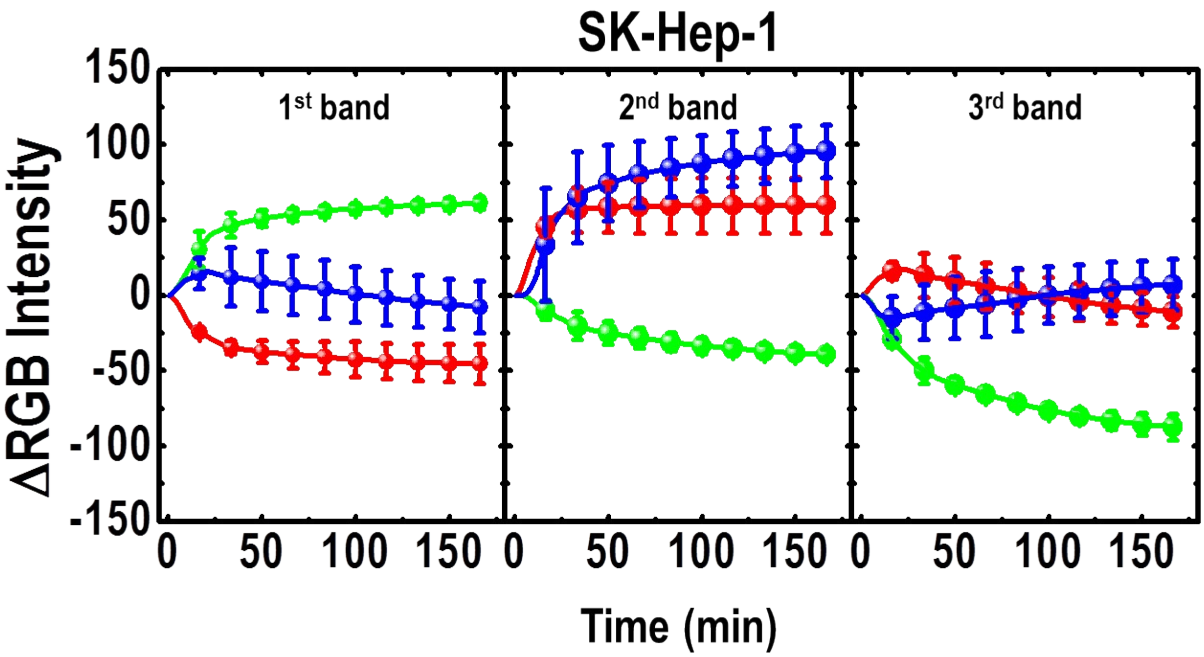

Figure S12. Real-time RGB color profiles of the M-13 bacteriophage-based photonic nose after exposure to SK-Hep-1 cell proliferation.

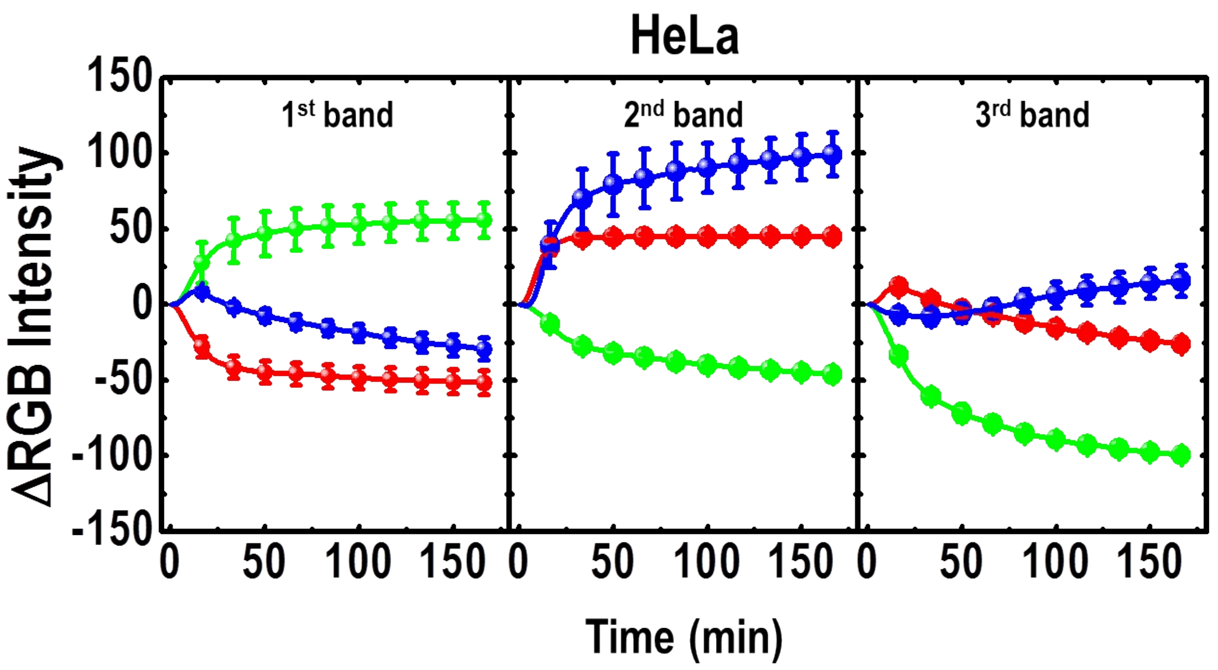

Figure S13. Real-time RGB color profile of the M-13 bacteriophage-based photonic nose after exposure to HeLa cell proliferation.

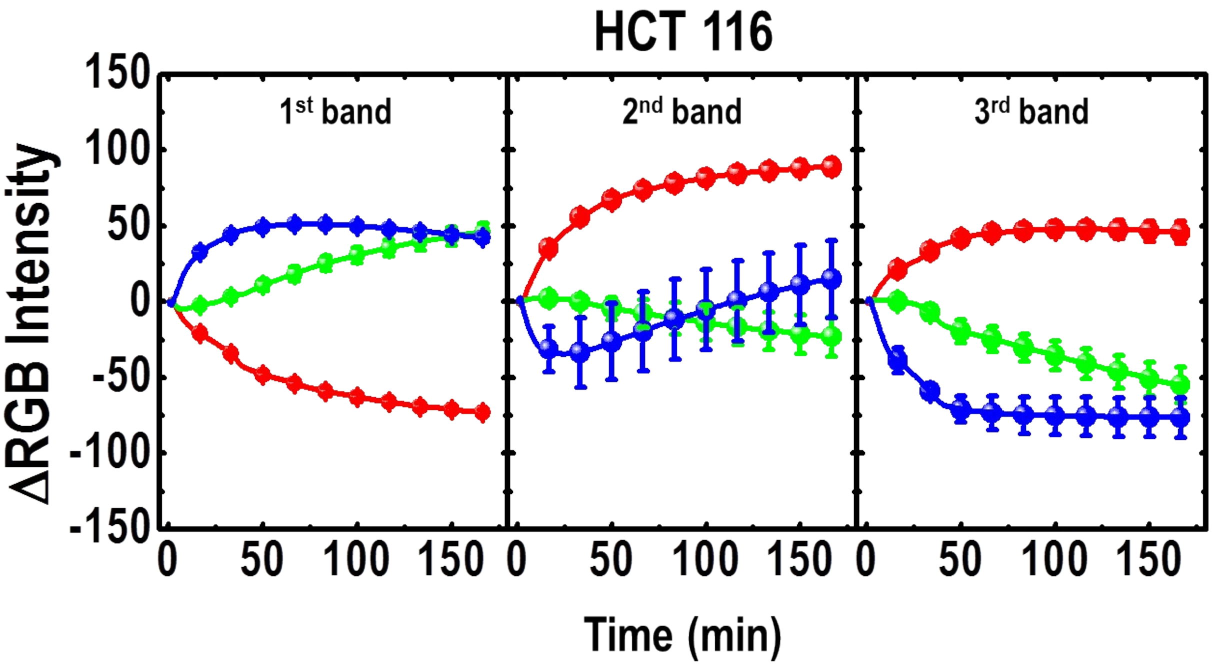

Figure S14. Real-time RGB color profile of the M-13 bacteriophage-based photonic nose after exposure to HCT116 cell proliferation.
